# Digital health implementation research across selected African countries: a bibliometric analysis of maternal health and infectious diseases with observations on precision medicine representation (2015–2025)

**DOI:** 10.3389/fdgth.2026.1831353

**Published:** 2026-06-23

**Authors:** Abayomi O. Agbeyangi, Sweeta Agrawal, Jose M. Lukose

**Affiliations:** 1 Walter Sisulu University, East London, South Africa; 2 Rama Devi Women’s University, Bhubaneswar, India

**Keywords:** Africa, bibliometric analysis, digital health, health systems, infectious diseases, maternal health, mHealth, precision medicine

## Abstract

**Background:**

Digital health technologies are increasingly recognised as important tools for strengthening healthcare delivery across African health systems. Despite rapid growth in digital health innovation, the implementation-oriented research landscape remains fragmented, with limited synthesis of the field's thematic structure, collaboration patterns, and evolving research priorities. This study maps the intellectual structure and thematic evolution of implementation-focused digital health research across selected African countries (South Africa, Kenya, Uganda, Nigeria, Ethiopia, Ghana, Tanzania and Rwanda).

**Methods:**

A bibliometric analysis was conducted on peer-reviewed publications indexed in Web of Science, Scopus, and PubMed between 2015 and 2025. Records were retrieved using a structured Boolean search strategy combining digital health, implementation, geographic, clinical-domain, care-setting, and outcome-related terms. The search captured implementation-oriented publications with explicit references to selected African countries. Following screening and deduplication, 440 publications were included. Analyses were performed using the Bibliometrix R package and included performance analysis, co-word analysis, thematic mapping, collaboration-network analysis, and temporal trend analysis.

**Results:**

Digital health research across the selected African countries expanded substantially during the study period, particularly after 2020. Research activity was strongly dominated by mobile-health-supported interventions targeting maternal health, HIV care, tuberculosis adherence, and primary healthcare delivery. Thematic mapping identified “mobile health–antenatal care–mobile phone” as a motor theme, while “mHealth–HIV–Kenya” emerged as a broadly connected but conceptually fragmented basic theme. Telemedicine-related research appeared as a specialised niche theme, whereas tuberculosis-focused digital adherence interventions occupied an emerging-or-declining thematic position. Keyword trend analyses further indicated a transition from feasibility-oriented studies toward implementation-focused and system-oriented digital health research. Precision medicine and rare-disease terminology did not form distinct thematic clusters within the retrieved corpus.

**Conclusions:**

Implementation-oriented digital health research across the selected African countries is undergoing rapid expansion, with increasing emphasis on scalable mobile health systems and integrated healthcare delivery. However, thematic fragmentation, limited interoperability, and the absence of longitudinal system-level evaluation remain important challenges.

## 1 Introduction

Digital health technologies have emerged as a key component of health system transformation, especially in low and middle-income countries (LMICs), by transforming health services and how patients access them, as well as health system clinical workflows (1–3). Mobile health (mHealth), telemedicine, electronic health records, and digital decision-support systems are being used more and more to overcome persistent problems with access to care, quality of services, continuity of care, and health workforce shortages (4–7). Digital technologies have been encouraged as scalable interventions in many contexts in Africa, characterised by limited resources in health systems and a high disease burden (8). Globally, digital health has been recognised as a key enabler of health system strengthening and universal health coverage. The Global Strategy on Digital Health 2020–2027 from the World Health Organisation (WHO) emphasises the need for sustainable digital health integration into national health systems through interoperable digital ecosystems, digital health governance frameworks, and evidence-informed digital health implementation (9). Similarly, studies by Agarwal et al. (10) and Sun et al. (2) emphasise the importance of improved evidence synthesis and system-level views to inform the responsible scale-up of digital health innovations, especially in LMICs with the greatest health system challenges (2, 10). Even with these calls globally, there has been limited mapping of digital health research trends from African contexts.

Digital health can be understood as a socio-technical ecosystem in which technologies, health actors, institutions, and data infrastructures interact to influence health outcomes and system performance (11). Rather than functioning as isolated tools, digital health interventions operate within complex systems where patient behaviours, clinical practices, and organisational processes mutually shape the design, implementation, and effectiveness of digital innovations (12, 13). This ecosystem perspective is particularly relevant in African health systems, where digital solutions are frequently integrated into heterogeneous care environments characterised by diverse health needs, varying infrastructure capacity, and uneven resource distribution (1, 14).

Over the past decade, digital health research in Africa has expanded rapidly, reflecting growing global and regional interest in technology-enabled health system strengthening. Early digital health initiatives on the continent were largely technology-driven and exploratory, focusing on feasibility assessments, infrastructure deployment, and proof-of-concept studies. These early efforts were often directed at priority public health challenges such as maternal and child health, HIV, tuberculosis, and malaria, using interventions including SMS-based communication, mobile data collection tools, and basic electronic medical records to support service delivery in rural and resource-constrained settings (15, 16). Over time, digital health research has matured to include more rigorous evaluation approaches, such as randomised controlled trials, economic evaluations, and implementation research, aimed at assessing effectiveness, scalability, and sustainability (12, 17).

More recently, the African digital health landscape has shifted towards implementation-focused and equity-oriented approaches. Research increasingly emphasises human-centred design, population-specific interventions, and contextually adapted technologies targeting women, children, adolescents, and individuals living with infectious or chronic diseases (18). In addition to maternal and infectious disease care, emerging literature has begun to explore applications in areas such as digital diagnostics and precision medicine, though these remain peripheral within the implementation-focused corpus and are not consistently represented using rare-disease-specific terminology (1, 7).

Country-level evidence further illustrates the diversity and depth of digital health implementation across the continent. Digital clinical decision-support tools and mHealth programs are used in South African PHCs to aid adherence to HIV treatment and maternal care coordination, but have faced many implementation difficulties, including issues with reliability and the readiness of infrastructure and staff (19). Mobile health interventions have been extensively adopted in Kenya for delivering HIV pre-exposure prophylaxis, communicating with antenatal care, and supporting community health workers, and have been found to increase service uptake and engagement (20, 21). Interactive voice response systems and peer-navigation tools have been found to be effective for enhancing ART adherence with youth living with HIV in Uganda, demonstrating the potential of context-adapted digital tools in low-resource settings (15, 22). In Nigeria, digital communication via chatbots and SMS has been tested to enhance HIV prevention outreach and clinic attendance among hard-to-reach groups (11, 16, 17). These country-level experiences demonstrate the growing body of digital health implementation research in African health systems and the ongoing challenges of scalability, sustainability, and interoperability across systems.

Despite this rapid expansion, the digital health literature in African contexts remains fragmented across disciplines, disease domains, technologies, and geographic settings. Research contributions are dispersed across fields such as public health, medical informatics, information systems, and implementation science, limiting the ability to develop a coherent understanding of the intellectual structure and thematic evolution of the field (23, 24). While existing systematic reviews have synthesised evidence on the effectiveness of specific interventions or disease domains (25), they provide limited insight into the broader knowledge structure, collaborative networks, and emerging research trajectories shaping digital health scholarship in Africa. Bibliometric analysis offers a powerful methodological approach for addressing this gap. By applying science-mapping techniques to large bodies of scholarly literature, bibliometric methods enable the systematic examination of research performance, thematic development, and intellectual linkages across a field (26). Techniques such as co-word analysis, thematic mapping, and temporal trend analysis allow researchers to identify influential actors, map knowledge structures, and uncover emerging research directions (27).

Against this background, this study conducts a comprehensive bibliometric analysis of implementation-oriented digital health research in Africa from 2015 to 2025, focusing on applications in maternal health and infectious disease management while also examining the extent to which precision medicine vocabulary surfaces within the retrieved literature. In this study, the latter stream captures rare-disease-relevant digital care as represented in the indexed literature through precision medicine, digital diagnostics, and data-driven clinical decision support. By combining performance analysis with co-word and thematic mapping techniques, the study systematically maps the intellectual landscape and evolution of digital health scholarship within African health systems. Specifically, this study addresses the following research questions: **RQ1**: Who are the most influential authors, institutions, and countries, and how are collaboration networks structured, in digital health research on maternal health and infectious diseases in Africa? **RQ2**: What are the dominant research themes and technologies, and how have they evolved between 2015 and 2025, within digital health research focused on maternal health and infectious disease management in Africa? **RQ3**: Which thematic clusters represent emerging or underexplored areas for future digital health research in maternal health and infectious diseases in Africa, and to what extent does precision medicine vocabulary appear within the literature?.

By addressing these questions, this study makes these important contributions. First, the study provides one of the most extensive bibliometric syntheses of digital health scholarship focused on Africa, offering a systematic overview of publication trends, influential contributors, and collaborative networks across the field. Second, through co-word analysis and thematic mapping, the study reveals the core, emerging, and specialised research themes shaping digital health innovation in maternal health, infectious disease management, and underrepresented precision-medicine and rare-disease-relevant research. Third, the analysis highlights underrepresented areas in African digital health research, particularly cross-disease digital health systems and integrated care platforms, and notes that precision medicine and rare-disease terminology rarely surface in implementation-focused publications, a pattern that warrants attention. Fourth, by linking thematic developments with broader health system priorities, the study provides an evidence-informed roadmap for advancing interoperable, scalable, and equitable digital health ecosystems within African health systems. Fifth, these insights are particularly timely as governments, development partners, and global health organisations increasingly seek to scale digital health innovations, including mHealth and telemedicine, from pilot initiatives towards sustainable system-level implementation.

## 2 Methods

### 2.1 Research design

This study employed a bibliometric research design to systematically map and synthesise the academic landscape of digital health research in Africa, with a particular focus on applications related to maternal health, infectious diseases, and underrepresented precision-medicine and rare-disease-relevant research. Bibliometric analysis provides a quantitative approach for examining large bodies of scholarly literature, enabling the identification of publication trends, influential contributors, collaborative networks, and thematic developments within a research field (26).

While narrative systematic reviews and scoping reviews usually yield a qualitative or semi-quantitative synthesis of evidence in a specific population, intervention, and outcome, bibliometric methods can be used to quantitatively analyse research productivity, intellectual structure and the development of scientific knowledge in interdisciplinary areas (27). Bibliometric analysis differs from systematic reviews in that it is a macro-level analysis of the patterns of scholarly output, collaboration, and thematic development within an entire research field.

This study uses bibliometric performance analysis and science-mapping methods to give a comprehensive view of the digital health scholarship pathway and its thematic organisation in selected African countries. The analytical scope mirrors the scope of the applied search strategy, and the results presented should be interpreted as a representation of the defined sample of the literature and not as the entirety of African digital health research.

### 2.2 Data sources and search strategy

To ensure comprehensive coverage of the relevant literature, bibliographic records were retrieved from three major academic databases: Scopus, PubMed, and the Web of Science Core Collection. These databases were selected for their broad coverage of peer-reviewed literature across clinical medicine, public health, digital health, and interdisciplinary research. Using multiple databases reduces the risk of single-source bias and enhances the representativeness of bibliometric analyses (26, 27). All database searches were conducted between 2 and 6 March 2026.

A Boolean search strategy was constructed to find peer-reviewed publications that focused on the implementation of digital health applications in African health systems. The approach was to integrate six concept blocks that are connected with Boolean operators: digital health technologies, geographic context, clinical domain, implementation focus, care setting, and health outcomes. This is a structure intended to obtain publications that explicitly focus on implementation aspects of digital health in specific geographic and clinical settings. Due to this design, the retrieved data set may not include publications that already cover digital health in African contexts but do not employ ‘implementation science’ terms, nor do exploratory/feasibility studies that preceded the introduction of implementation science terms (See Supplementary Appendix for full search strings used).

The search was restricted to publications from 2015 to 2025, reflecting a decade of accelerated digital health innovation and implementation across African health systems. Only peer-reviewed journal articles published in English were included to ensure consistency, accessibility, and quality of the dataset.

The search strategy was fairly broad and included the term precision medicine, but it did not use more specific rare-disease and genomics terminology like orphan disease, sickle cell disease, pharmacogenomics, or next-generation sequencing. Thus, the dataset obtained is mostly conceptual in the digital health implementation domain rather than the broader landscape of precision medicine and rare-disease research in African contexts. The geographic concept block incorporated the following countries explicitly: South Africa, Kenya, Uganda, Nigeria, Ethiopia, Ghana, Tanzania and Rwanda. These countries are important sources of digital health implementation research in sub-Saharan Africa, but the countries listed do not include all African countries with active digital health research programmes. The geographic search block used does not explicitly include countries such as Egypt, Morocco, Senegal, Zimbabwe, Malawi, Cameroon, and the Democratic Republic of Congo. Consequently, publications from these and other countries that did not co-mention the selected countries in the title, abstract, or keywords would not have been retrieved. This geographic coverage is not full continental coverage, but represents a careful selection of countries for implementation-oriented research.

Taken together, the Boolean query structure and the defined country list mean that the dataset captures a specific, bounded segment of the African digital health literature, namely, implementation-focused studies with explicit geographic anchoring in the selected countries. Therefore, the findings, thematic patterns and identified research gaps must be seen within the small analytical scope of this study and not as a representation of all digital health research across Africa.

### 2.3 Data screening, deduplication, and consolidation

Bibliographic records retrieved from the three databases were exported and integrated using Biblioshiny, the web-based interface of the *bibliometrix* R package (27). Data consolidation and deduplication were performed using the *mergeDbSources()* function within the bibliometrix environment.

A two-stage deduplication procedure was applied. First, duplicate records were identified and removed based on exact DOI matching. Second, records without DOI identifiers were screened using normalised title and publication year matching, a widely recommended approach for bibliometric datasets compiled from multiple databases (26).

The initial dataset comprised 809 records retrieved across the three databases. Following automated deduplication, 369 duplicate entries were removed, resulting in a final dataset of 440 unique publications for bibliometric analysis.

**Figure 1:**
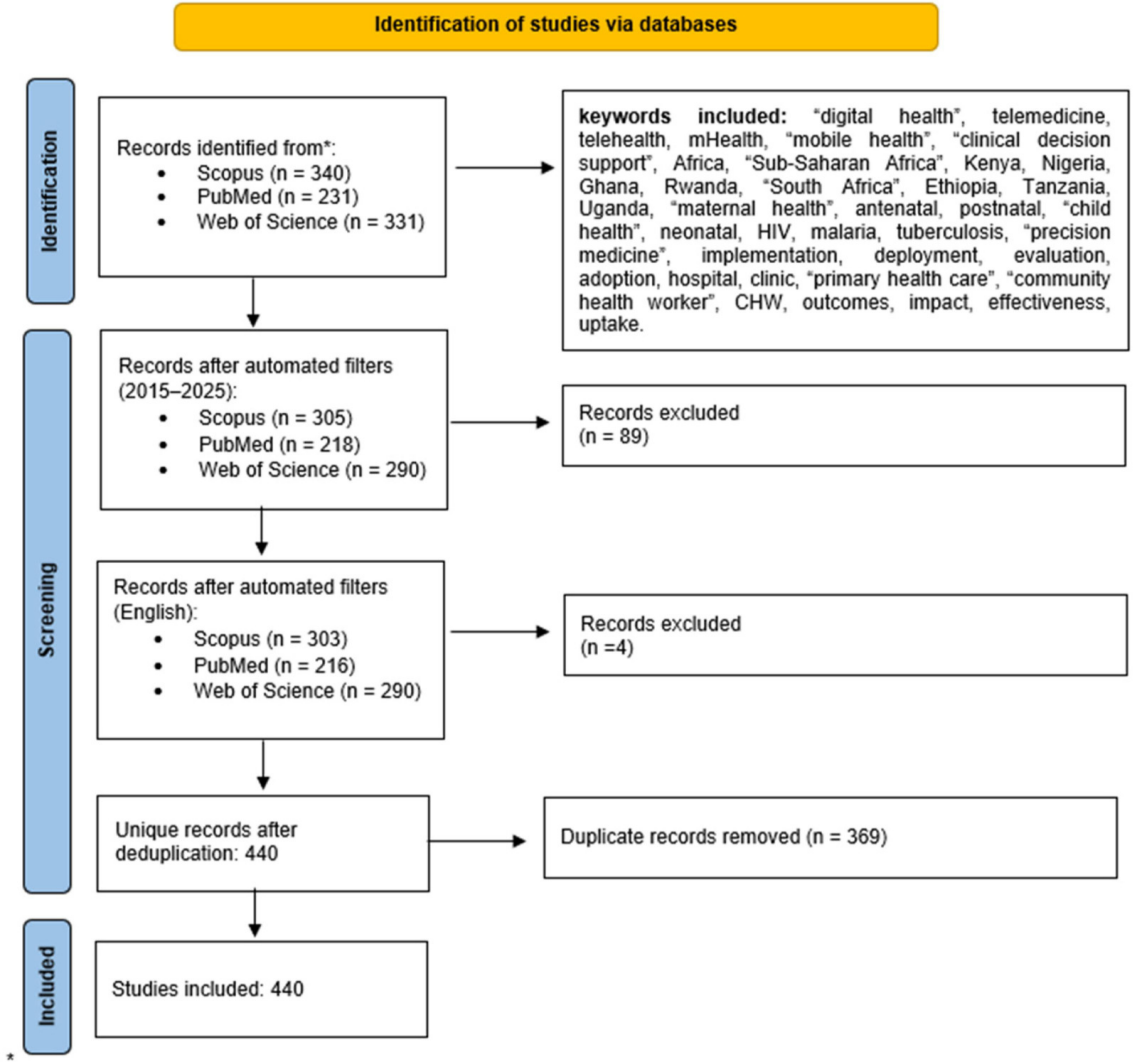
PRISMA flow diagram illustrating the identification, screening, deduplication, and final inclusion of publications used in the bibliometric analysis of digital health research across selected African countries between 2015 and 2025.

To enhance transparency and reproducibility, the screening and selection process followed the PRISMA 2020 (Preferred Reporting Items for Systematic Reviews and Meta-Analyses) framework, which is increasingly recommended for structured evidence synthesis, including bibliometric studies (28). The study selection process is illustrated in Figure 1.

### 2.4 Data analysis and tool selection

Bibliometric analyses were conducted using Biblioshiny, the graphical interface of the *bibliometrix* R package, which enables transparent, reproducible, and comprehensive bibliometric and science-mapping analyses. The analytical procedure was conducted in two stages. First, a performance analysis was performed to examine the structural characteristics of the literature. This included the assessment of annual publication trends, most productive journals, influential authors, institutional contributions, country-level productivity, and international collaboration networks (29). Second, science-mapping techniques were applied to explore the intellectual and conceptual structure of the research field. Specifically, co-word analysis and thematic mapping were used to identify dominant research themes, emerging topics, and the temporal evolution of digital health research across maternal health, infectious disease management, and underrepresented precision-medicine and rare-disease-relevant research in African contexts.

These combined analytical approaches enable a comprehensive understanding of the field's performance and knowledge structure, thereby providing insights into the development, fragmentation, and future directions of digital health research in Africa.

### 2.5 Science mapping techniques

To explore the conceptual structure and thematic evolution, this study employed science-mapping techniques within the *Bibliometrix* analytical framework. Science mapping enables the visualisation and quantitative analysis of relationships between keywords, publications, and research themes, thereby revealing the intellectual structure and development of a research field (27, 30, 31).

First, co-word analysis was conducted to examine the co-occurrence patterns of author keywords within the dataset. Co-word analysis identifies relationships between frequently occurring terms, allowing the detection of dominant research topics and conceptual linkages across the literature. By analysing the frequency with which keywords appear together within publications, this technique provides insights into the conceptual organisation of the field and the relative prominence of specific research themes (31). Second, thematic mapping was applied to classify research themes by level of development and importance within the field. The thematic map generated by *Bibliometrix* is based on two key dimensions: centrality, which measures the degree of interaction between a theme and other themes within the research field, and density, which reflects the internal development and cohesion of a particular theme. Based on these two dimensions, themes were classified into four quadrants: motor themes (high centrality and high density), which are well developed and central to the field; niche themes (low centrality and high density), which are specialised and internally developed; basic themes (high centrality and low density), which are important but less developed; and emerging or declining themes (low centrality and low density), which are weakly developed and peripheral. Third, thematic evolution analysis was performed to examine how research topics have changed over time within the digital health literature. This analysis enables the identification of thematic transitions and the emergence of new research directions across different time periods. By tracking the evolution of keyword clusters, thematic evolution analysis provides insight into how digital health research in Africa has shifted in focus across domains such as maternal health, infectious disease management, and underrepresented precision-medicine and rare-disease-relevant research.

Together, these science-mapping techniques provide a comprehensive understanding of both the conceptual structure and the temporal development of digital health scholarship in Africa. The combined use of performance analysis and science mapping enables a robust examination of research productivity, intellectual organisation, and emerging thematic trends within the field.

## 3 Results

This section presents the bibliometric findings on digital health research in Africa from 2015 to 2025, focusing on research productivity, influential contributors, collaboration patterns, and the field's thematic evolution. The analysis provides insights into how digital health scholarship has developed across maternal health, infectious diseases, and emerging areas such as precision and underrepresented precision-medicine and rare-disease-relevant research, highlighting both the structural organisation and the evolving priorities of the research landscape.

### 3.1 Descriptive overview of the dataset

Table 1 summarises the key bibliometric characteristics of the dataset. Following screening and deduplication, 440 publications published between 2015 and 2025 were retained for analysis. These publications represent implementation-oriented digital health research with explicit mentions of selected African countries, as defined by the applied Boolean search strategy. These publications were distributed across 148 academic sources**,** reflecting the interdisciplinary nature of digital health research spanning public health, clinical medicine, health informatics, and implementation science. The dataset demonstrates a strong growth trajectory, with an annual publication growth rate of 15.75%, indicating rapidly increasing scholarly attention to digital health implementation in African health systems. The average document age of 4.78 years highlights the literature's contemporary nature, while the mean citation rate of 13.77 citations per article reflects a steadily expanding evidence base.

**Table 1:** Descriptive characteristics and key bibliometric indicators of the analysed dataset of digital health publications across selected African countries(2015–2025).

Metric	Value
Timespan of the sample	2015–2025
Sources	148
Documents	440
Annual growth rate (%)	15.75
Average citations per document	13.77
Document average age (years)	4.78
Authors	3,676
Single-authored documents	3
Co-authors per document	10.4
International co-authorship (%)	57.05
Author keywords	1,170
Keywords Plus	1,846

Early studies in the dataset focused primarily on foundational digital health applications, including SMS-based communication systems and electronic medical record platforms deployed to support maternal care and health service delivery in rural contexts (22). Over time, the literature evolved towards more rigorous evaluation approaches, including randomised controlled trials, cost-effectiveness analyses, and system-level implementation studies assessing the impact of digital health interventions across African healthcare systems.

Authorship patterns further highlight the field's collaborative nature. The dataset includes 3,676 authors, with only three single-authored publications. On average, each article involved 10.4 co-authors, and 57.05% of publications included international co-authorship, reflecting the interdisciplinary and globally collaborative character of digital health implementation research.

In terms of document types, the dataset predominantly consists of original research articles (*n* = 376) and review papers (*n* = 34), with a smaller number of clinical trial protocols and early-access publications. The dominance of empirical studies suggests that the field has progressed beyond exploratory stages towards evidence-driven evaluation of intervention effectiveness and health outcomes, particularly in maternal health, HIV and tuberculosis care, and child health interventions.

### 3.2 Growth of scientific production

**Figure 2:**
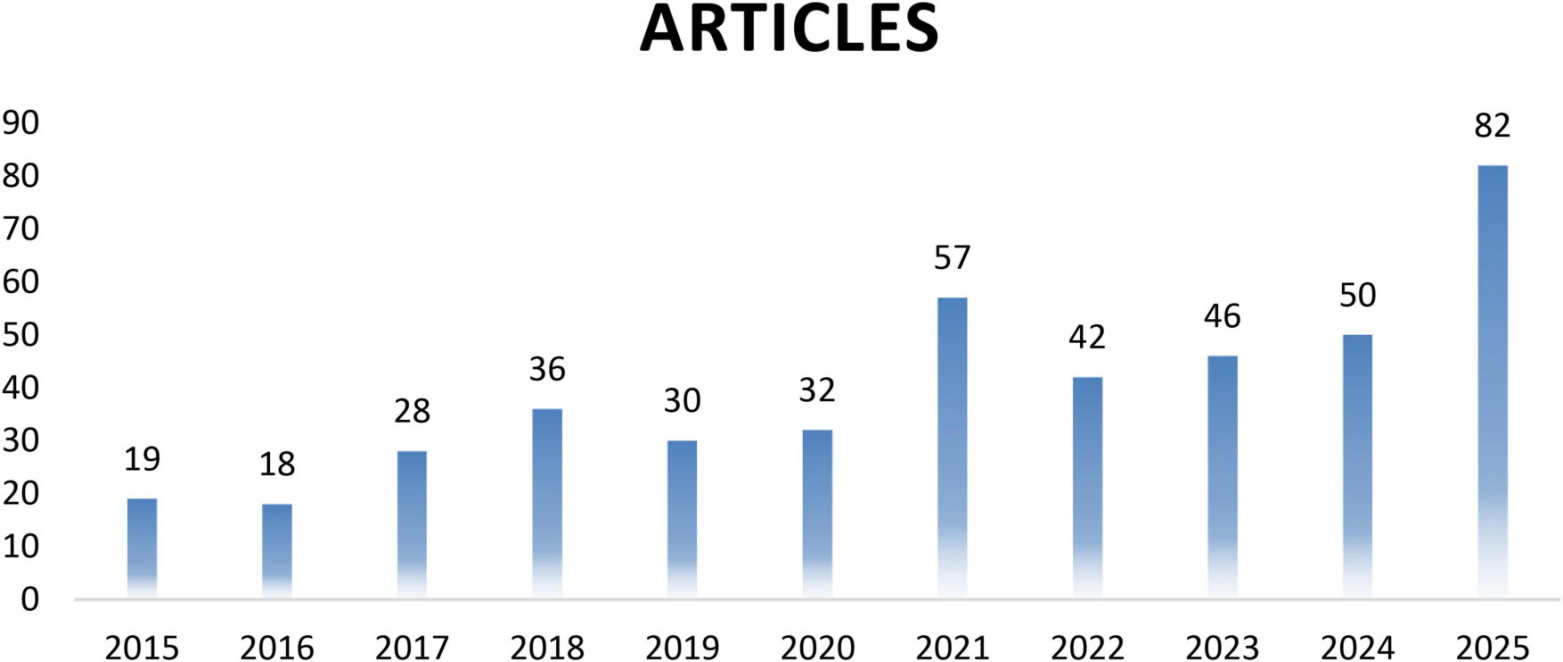
Annual scientific production of implementation-oriented digital health publications across selected African countries between 2015 and 2025.

The annual scientific output of digital health publications in the countries of Africa selected is shown in Figure 2 from 2015 to 2025. The number of publications grew steadily over the course of the study, especially since 2020. The highest annual output occurred in (*n* = 82), reflecting continued growth of implementation-based digital health research in health systems in Africa.

**Figure 3:**
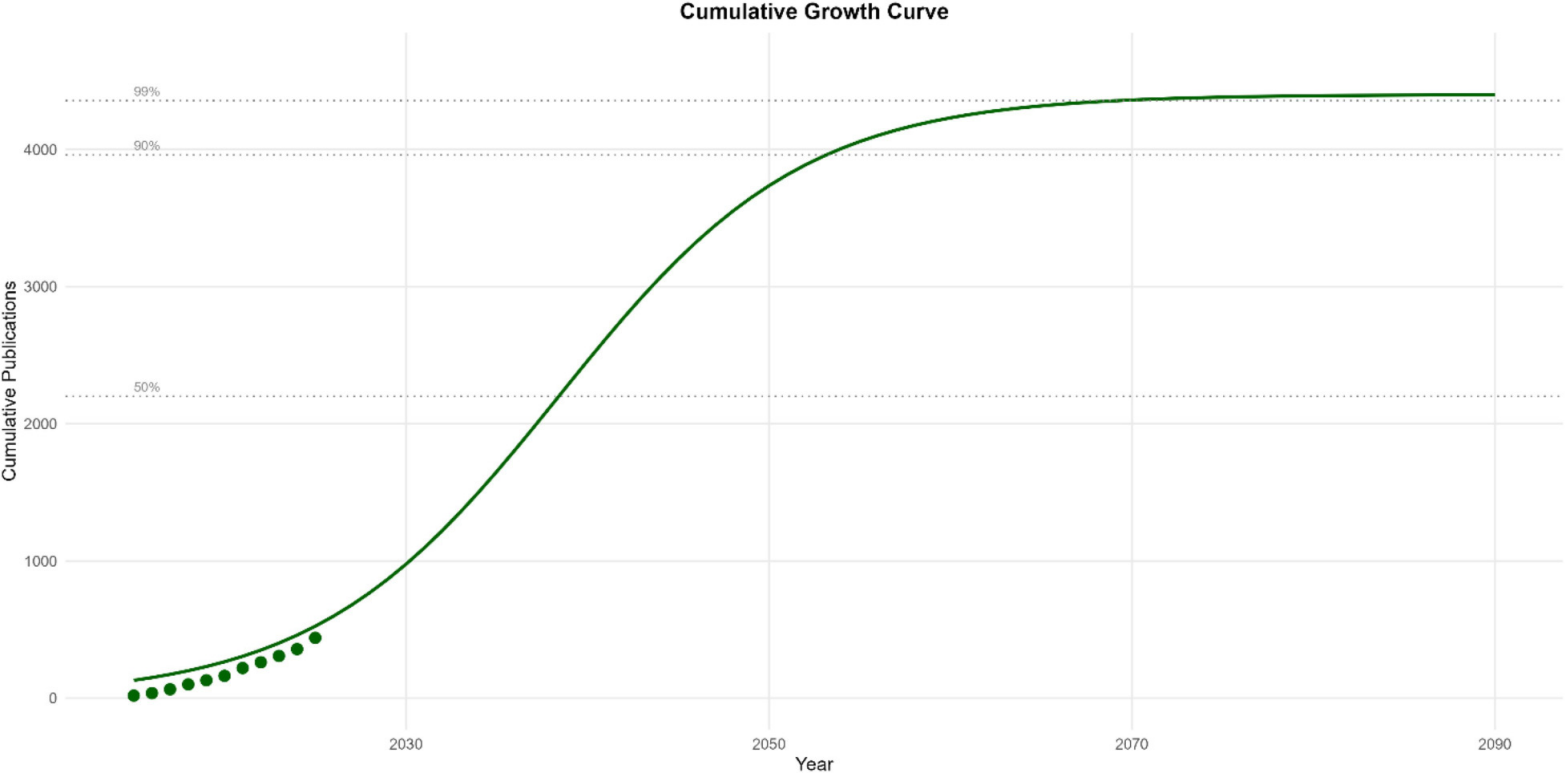
Exploratory cumulative growth curve of digital health publications across the selected African countries between 2015 and 2025, fitted using a logistic growth model. The curve is presented as an illustrative representation of publication dynamics and should not be interpreted as a definitive predictive forecast.

For a further analysis of the publication dynamics, a fitted logistic growth model was used in this study (Figure 3). The overall growth curve suggests that publication activity has continued to rise over time, reflecting the trend of increasing digital health implementation research in the countries included in this study across Africa. The relatively short time span of the data and the changing nature of the field should be taken into account when considering long-term saturation levels and future growth curves. Accordingly, the model is presented as an exploratory representation of publication dynamics rather than a definitive predictive forecast.

### 3.3 Influential Authors, Journals, and Institutions

Table 2 presents the most influential authors contributing to digital health implementation research in Africa. Scholars such as Keshet Ronen, Grace John-Stewart, and Edward J. Mills rank among the most productive authors in terms of publication output and citation impact. However, when considering time-adjusted scholarly influence, Amnesty LeFevre demonstrates the highest m-index (1.17), indicating rapid growth in scholarly impact within a shorter research career span. This result suggests that the field is shaped by a combination of highly established senior scholars and emerging researchers with rapidly increasing influence, reflecting the dynamic and evolving nature of digital health research.

**Table 2:** Most productive authors contributing to digital health research across selected African countries, ranked by publication output and citation-based impact indicators.

Rank	Author	Articles	Total Citations (TC)	h-index	Years Active	m-index
1	Keshet Ronen	9	105	6	8	0.75
2	David R. Bangsberg	8	200	6	10	0.60
3	Grace John-Stewart	8	97	5	8	0.63
4	Edward J. Mills	8	295	7	9	0.78
5	Jennifer A. Unger	8	99	5	8	0.63
6	Amnesty LeFevre	7	139	7	6	1.17
7	Sandra I. McCoy	7	102	5	6	0.83
8	John Kinuthia	7	90	4	8	0.50
9	Edwin D. Charlebois	6	69	4	10	0.40
10	Alain Labrique	6	80	5	6	0.83

Table 3 identifies the most prominent journals publishing digital health research in African contexts. PLOS ONE emerges as the most influential publication outlet, with 30 articles, the highest h-index (23), and 532 total citations, reflecting its role as a central interdisciplinary platform for digital health research. Other major sources include BMC Health Services Research and BMJ Open, both of which emphasise health systems research and policy-oriented scholarship. Notably, journals within the JMIR family (JMIR mHealth and uHealth, JMIR Research Protocols, and JMIR Formative Research) also contribute substantially to the literature, highlighting the importance of digital medicine and implementation science frameworks. The distribution of sources indicates that digital health research in Africa is primarily concentrated in open-access, methodologically rigorous, and interdisciplinary journals.

**Table 3:** Leading publication sources in digital health research across selected African countries, ranked by number of publications and citation-based performance metrics.

Rank	Source	Articles (NP)	h-index	g-index	m-index	Total Citations (TC)	Publication Year (start)
1	PLOS ONE	30	15	22	1.25	532	2015
2	BMC Health Services Research	24	12	18	1.00	330	2015
3	BMJ Open	24	11	21	1.00	469	2016
4	JMIR Research Protocols	21	6	8	0.50	76	2015
5	JMIR mHealth and uHealth	19	10	14	1.11	213	2018
6	JMIR Formative Research	16	6	7	1.00	67	2021
7	Trials	16	9	13	0.75	187	2015
8	BMC Pregnancy and Childbirth	15	7	11	0.64	141	2016
9	BMC Public Health	10	7	10	0.58	246	2015
10	Journal of Medical Internet Research	9	5	9	0.42	150	2015

Table 4 presents the leading institutional affiliations contributing to digital health research in Africa. The University of Washington ranks first with 78 publications, followed by Makerere University (32) and the University of the Witwatersrand (33). African institutions, particularly those in South Africa and Uganda, are strongly represented, reflecting growing regional research capacity. At the same time, institutions from the Global North, including Johns Hopkins University and the London School of Hygiene & Tropical Medicine, continue to play important roles through collaborative research partnerships. Overall, the institutional landscape reflects a hybrid research ecosystem characterised by strong North–South partnerships and increasingly prominent African research leadership.

**Table 4:** Top institutional affiliations contributing to digital health research across selected African countries based on publication output.

Rank	Affiliation	Country	Articles
1	University of Washington	United States	78
2	Makerere University	Uganda	39
3	University of the Witwatersrand	South Africa	38
4	University of Cape Town	South Africa	31
5	University of Ghana	Ghana	30
6	University of KwaZulu-Natal	South Africa	30
7	Johns Hopkins University	United States	29
8	London School of Hygiene & Tropical Medicine	United Kingdom	26
9	University of Washington (duplicate affiliation variant)	United States	26
10	Mbarara University of Science and Technology	Uganda	24

University of Washington appears twice due to affiliation variants in the bibliographic dataset.

### 3.4 Country-Level Scientific Production and Collaboration

Table 5 summarises country-level contributions within the retrieved dataset. It should be noted that country representation in this analysis is shaped by the geographic concept block of the search strategy, which included ten explicitly named countries. Countries not named in the search block are not represented in the dataset, regardless of their actual research output. The findings, therefore, reflect relative productivity among the selected countries rather than a comprehensive ranking of African nations by digital health research contribution.

**Table 5:** Top countries represented within the retrieved dataset of implementation-oriented digital health research across selected African countries.

Rank	Country	Articles	Articles %	SCP	MCP	Total Citations (TC)
1	USA	120	27.3	41	79	1,580
2	South Africa	55	12.5	27	28	804
3	United Kingdom	24	5.5	6	18	503
4	Uganda	28	6.4	12	16	304
5	Kenya	18	4.1	9	9	235
6	Ethiopia	31	7.0	19	12	282
7	Tanzania	14	3.2	6	8	117
8	Nigeria	18	4.1	14	4	133
9	Canada	10	2.3	5	5	366
10	Ghana	11	2.5	5	6	97

Country representation reflects the geographic concept block of the search strategy and does not constitute a comprehensive ranking of African nations by digital health research output.

The United States contributes the largest share of publications (27.3% of total output) and records the highest citation impact (TC = 1,580). Among African countries, South Africa emerges as the leading contributor, demonstrating both high publication output and citation impact. Other African countries, including Uganda, Kenya, Ethiopia, Nigeria, Tanzania, and Ghana, also demonstrate substantial research contributions, reflecting expanding regional research capacity.

The balance between single-country publications (SCP) and multi-country publications (MCP) indicates strong international collaboration across the field. Countries such as Canada and the United Kingdom demonstrate particularly high citation impact relative to publication volume, suggesting the influence of targeted collaborative contributions.

These findings reveal a globally interconnected research ecosystem in which African countries increasingly function as active knowledge producers rather than solely as research settings.

### 3.5 Author and country collaboration networks

**Figure 4:**
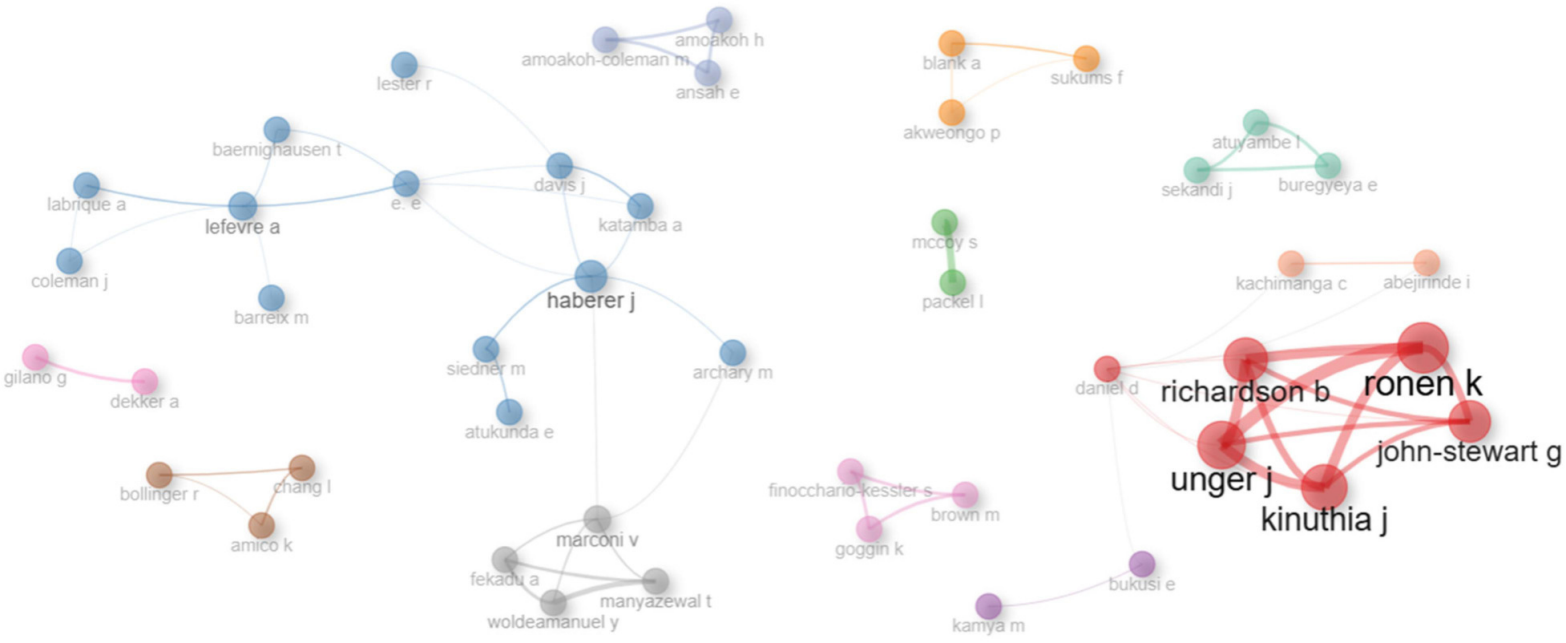
Author collaboration network showing co-authorship relationships among leading contributors to digital health research across selected African countries. Node size reflects publication volume; colours denote distinct research clusters identified through network analysis. Only authors with a minimum of two co-authored publications are displayed.

**Figure 5:**
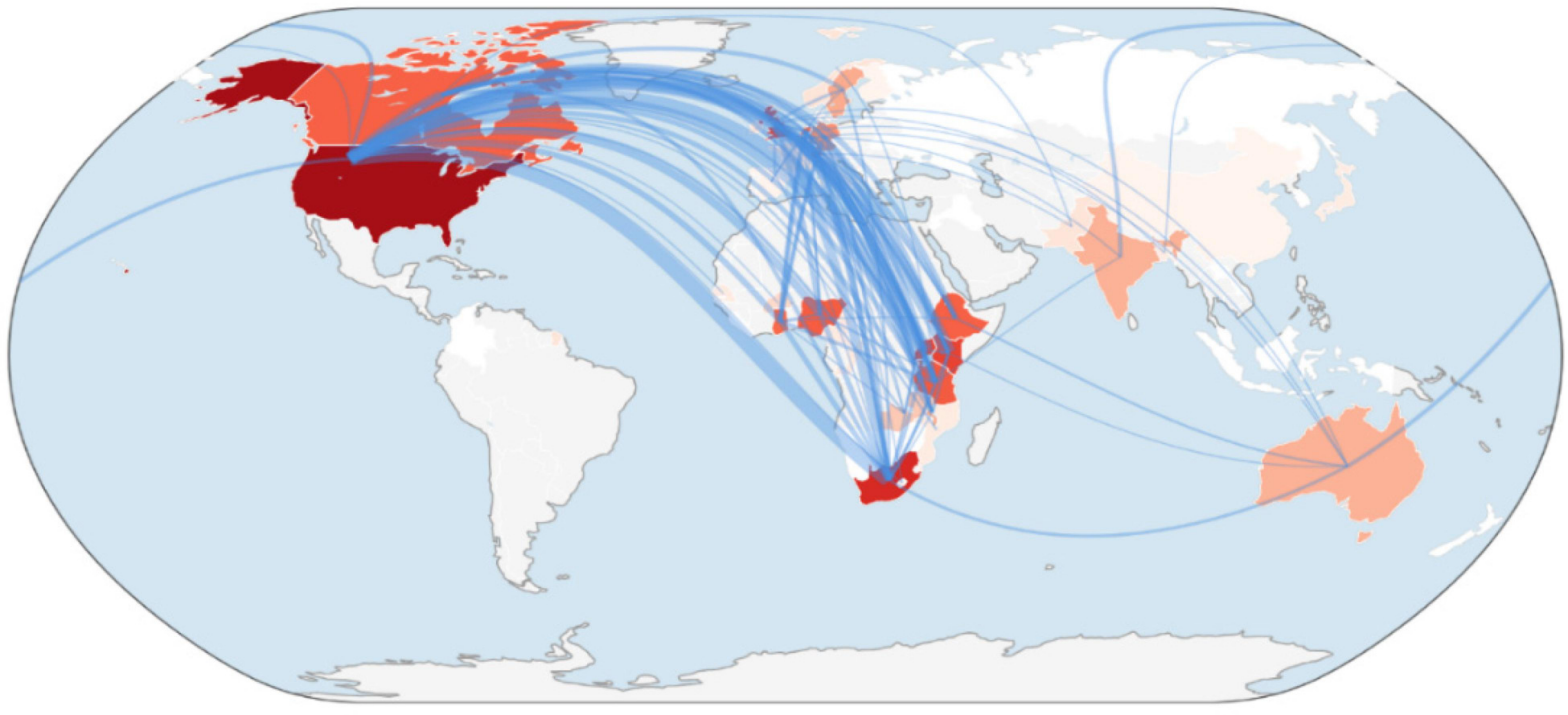
Country collaboration network illustrating international research partnerships in digital health research across selected African countries. Node size reflects total publication output; link thickness reflects the frequency of collaborative publications between countries. Colours denote distinct regional collaboration clusters.

Figures 4, 5 illustrate collaboration networks among authors and countries. The author collaboration network (Figure 4) reveals several dense research clusters rather than a single unified network. Prominent clusters are centred around highly productive authors, including Ronen, John-Stewart, Kinuthia, Unger, and Richardson**,** whose work primarily focuses on HIV, maternal health, and digital health implementation trials. Several authors, including Haberer, LeFevre, Davis, and McCoy, display high betweenness centrality, suggesting that they act as bridges connecting different research communities across clinical research, digital intervention design, and health systems evaluation.

The country collaboration network (Figure 5) highlights a highly globalised research environment. The United States occupies a central position, collaborating frequently with South Africa, Kenya, Uganda, and the United Kingdom. Notably, the network also reveals growing intra-African collaborations, particularly between South Africa, Kenya, Uganda, and Tanzania, indicating strengthening intra-African research partnerships.

### 3.6 Thematic evolution of digital health research

**Figure 6:**
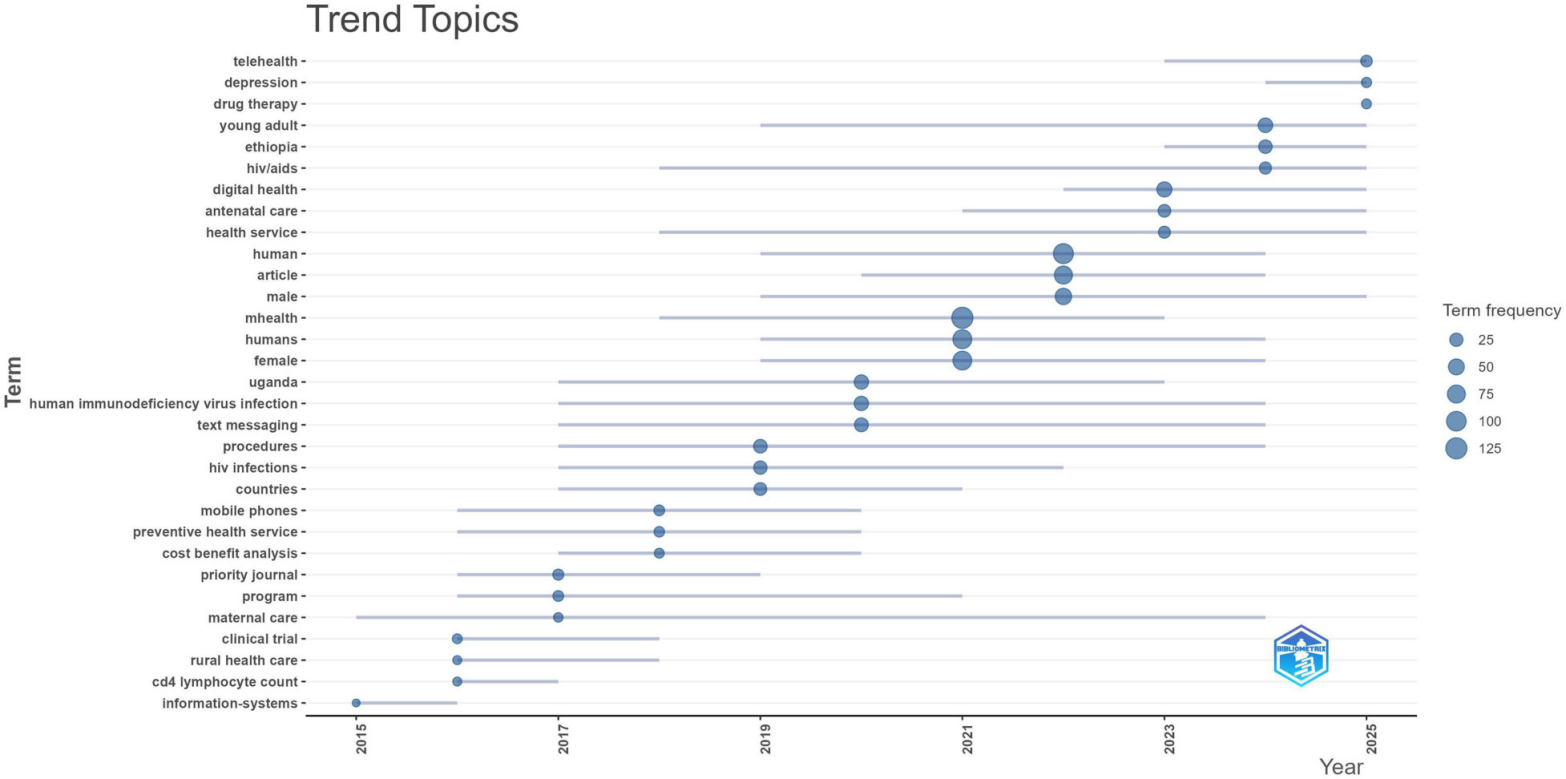
Trend topic analysis showing the temporal evolution of major research themes in digital health studies across selected African countries between 2015 and 2025.

Trend-topic analysis (Figure 6) demonstrates a clear chronological evolution of digital health research themes. Early research (2015–2016) focused primarily on health information systems, feasibility studies, and clinical validation of digital tools in low-resource settings. Between 2017 and 2019, the research agenda shifted towards implementation studies and disease-specific applications, particularly HIV-related digital interventions.

Since 2018, mHealth has emerged as the dominant research theme, reflecting the widespread adoption of mobile technologies for healthcare delivery. More recent years (2022–2025) show growing thematic diversification, with emerging topics including telehealth, antenatal care, mental health, and integrated digital health systems.

**Figure 7:**
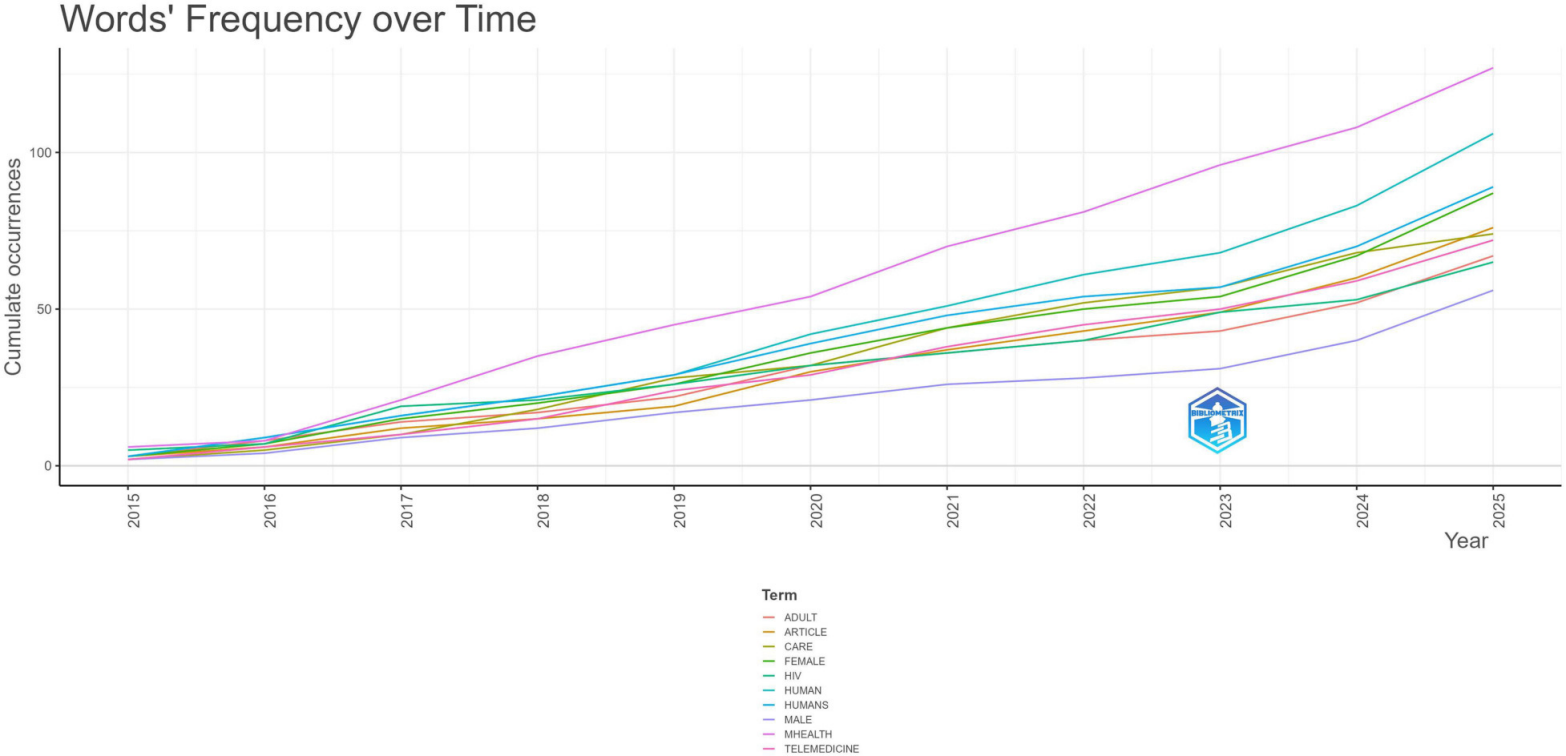
Temporal evolution of high-frequency keywords in digital health research across selected African countries from 2015 to 2025.

Figure 7 illustrates the temporal growth of high-frequency keywords. The term mHealth demonstrates the most dramatic increase, rising from six occurrences in 2015 to 127 in 2025, confirming its central role in digital health implementation research. Similarly, telemedicine shows steady growth, increasing from two occurrences in 2015 to 72 in 2025, reflecting increasing adoption of remote care technologies. Disease-specific terms such as HIV also show consistent growth, reinforcing the continuing importance of infectious disease management within the digital health research agenda.

### 3.7 Thematic structure of the field

**Figure 8:**
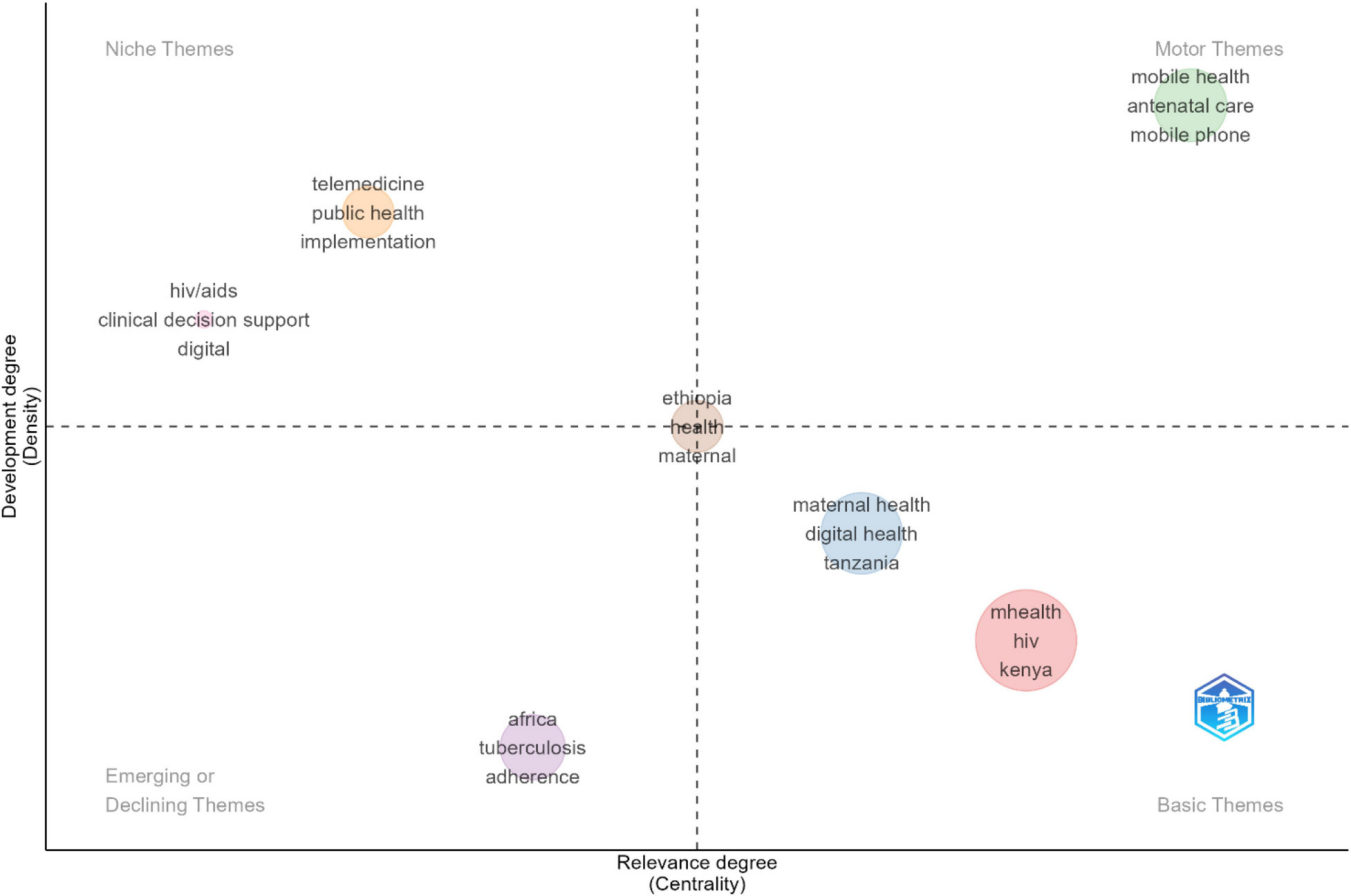
Thematic map of implementation-oriented digital health research across selected African countries, based on callon centrality and density, generated using author keywords. Colours denote distinct thematic clusters: motor themes (upper right), niche themes (upper left), basic themes (lower right), and emerging or declining themes (lower left).

The thematic map (Figure 8) visualises the intellectual landscape of African countries where implementation-oriented digital health research takes place. The analysis was carried out with the author keywords, instead of the general database keywords, to increase the thematic specificity and decrease the indexing artefacts. Mobile-health-related studies have a significant influence on thematic development. The ‘mobile health–antenatal care–mobile phone’ cluster is a motor theme, reflecting a well-established and highly interconnected research domain focused on antenatal care interventions. The “mHealth–HIV–Kenya” cluster falls in the basic-theme quadrant and has a moderate level of relevance to the overall theme, but a relatively weak level of internal coherence, indicating a less thorough conceptual integration across implementation clusters.

The “telemedicine–public health–implementation” cluster appears to be a “niche” theme, where the internal development is high and the linkage with other research areas is relatively low. By contrast, the “tuberculosis–adherence–Africa” cluster is in the emerging-or-declining quadrant, with relatively few thematic elements developed and integrated into the broader digital health literature. Maternal health, HIV care, and implementation-oriented mobile health interventions are of particular interest within the literature. Precision medicine and rare-disease terminology did not form distinct thematic clusters within the dataset, consistent with the implementation-oriented scope of the search strategy.

These trends are summarized in Table 6, which serves as a research agenda for the future that highlights the need for theory-driven frameworks for implementation, interoperable digital infrastructure, an integration of multiple diseases, and studies assessing the digital infrastructure at the population level over time.

**Table 6:** Future research agenda derived from thematic cluster analysis of digital health research across selected African countries.

Thematic Cluster	Structural Insight	Key Research Gaps	Priority Directions for Future Research
Mobile Health–Antenatal Care–Mobile Phone	High centrality and density (motor theme)	Limited long-term evaluation and interoperability across maternal health systems	Conduct longitudinal studies on maternal mHealth outcomes; strengthen interoperability between mobile platforms and national health systems
mHealth–HIV–Kenya	High centrality but lower density (basic theme)	Fragmented implementation frameworks; inconsistent outcome measures	Develop theory-driven implementation models integrating behavioural science, implementation science, and health systems approaches; standardise evaluation metrics
Maternal Health–Digital Health–Tanzania	Broad thematic relevance with moderate development	Limited comparative and cross-program integration	Expand comparative studies linking maternal health platforms with HIV, adolescent, and primary healthcare services
Telemedicine–Public Health–Implementation	High internal cohesion but weaker network integration (niche theme)	Limited integration with broader digital health ecosystems	Examine interoperability standards, governance frameworks, and hybrid care models integrating telemedicine with broader digital infrastructures
Tuberculosis–Adherence–Africa	Emerging or weakly integrated thematic area	Disease-specific silos and limited cross-disease integration	Investigate integrated digital adherence platforms linking tuberculosis, HIV, and chronic disease management
Precision Medicine and Rare-Disease Research	No distinct thematic cluster identified	Limited visibility within implementation-focused digital health literature	Develop dedicated bibliometric and implementation studies using explicit rare-disease and genomic medicine terminology

### 3.8 Keyword co-occurrence network

**Figure 9:**
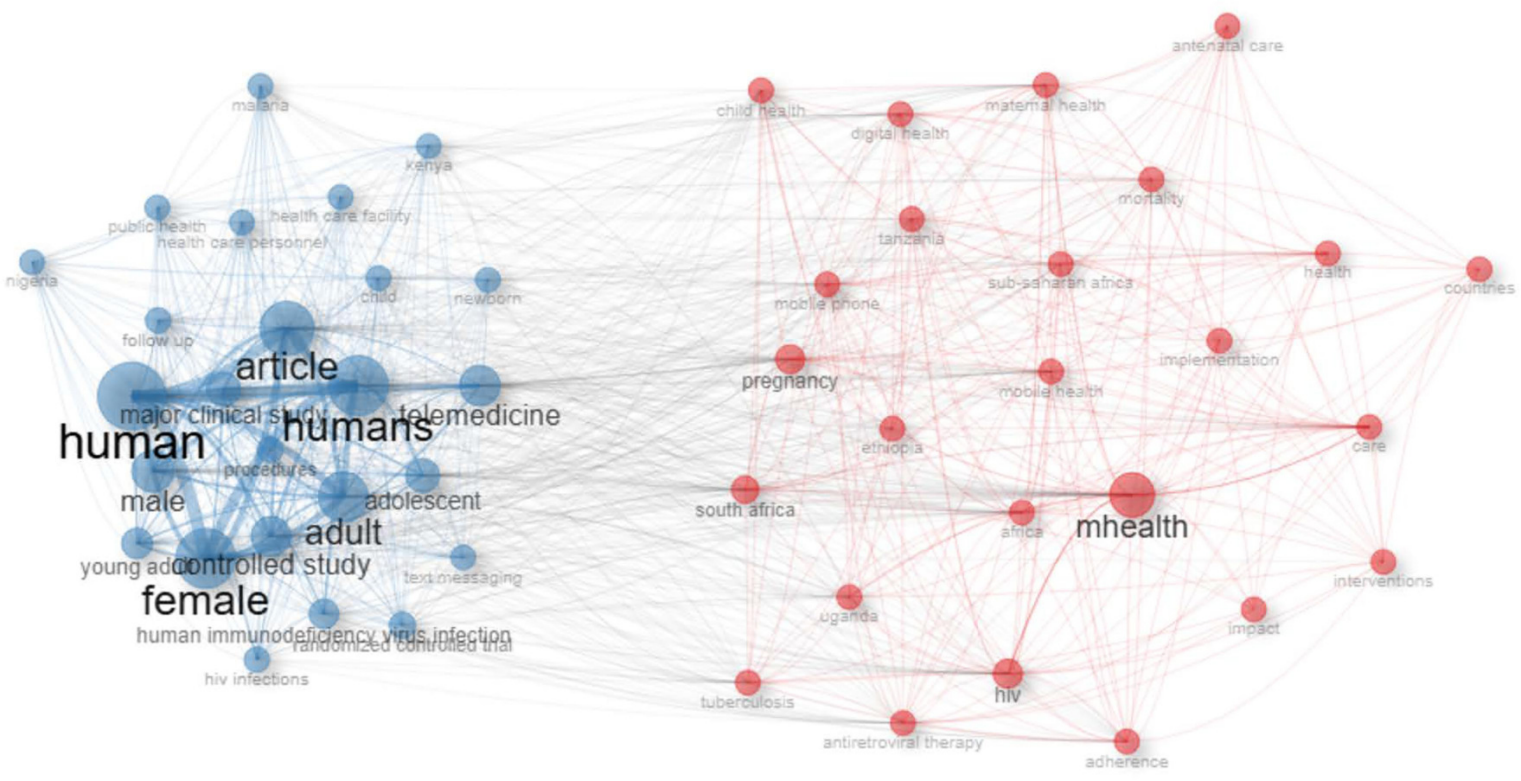
Keyword co-occurrence network illustrating the conceptual relationships and dominant thematic clusters within implementation-oriented digital health research across selected African countries. Colours denote two primary thematic clusters: implementation-oriented digital health applications centred on mHealth (cluster 1) and population and methodological dimensions including study designs and demographic descriptors (cluster 2). Node size reflects keyword frequency; link thickness reflects co-occurrence strength.

The keyword co-occurrence network (Figure 9) reveals two major thematic clusters. The first cluster centres on implementation-oriented digital health applications, with mHealth functioning as the central node linking HIV care, maternal health interventions, tuberculosis treatment adherence, and country-specific research contexts. The second cluster emphasises population and methodological dimensions, including demographic descriptors and experimental research designs such as randomised controlled trials and follow-up studies.

Together, these clusters demonstrate a mature research field characterised by strong connections between disease-focused digital health interventions and rigorous clinical evaluation frameworks.

### 3.9 Integration of temporal and thematic patterns

The integration of trend-topic analysis with the thematic map provides strong evidence of the progressive maturation of digital health research in Africa. Early research focused primarily on technological feasibility, whereas more recent studies increasingly emphasise large-scale implementation, patient-centred outcomes, and integrated health system applications.

Overall, the bibliometric results indicate that digital health research in Africa has evolved from exploratory technology development towards a mature field focused on scalable, population-centred digital health systems.

## 4 Discussion

This study presents a comprehensive bibliometric synthesis of digital health research in Africa between 2015 and 2025, offering insights into the field's intellectual structure, thematic evolution, and emerging research priorities. By combining performance analysis with science-mapping techniques, the study reveals a rapidly expanding research landscape characterised by strong international collaboration, increasing methodological maturity, and evolving thematic priorities centred on maternal health, infectious diseases, and emerging digital health applications. The findings suggest that digital health research in African contexts is transitioning from technology-focused experimentation towards integrated, implementation-driven health system applications (2, 27, 34). This shift reflects broader global trends in digital health research, where the emphasis is increasingly placed on scalability, sustainability, and health system integration rather than solely on technological feasibility (35, 36).

### 4.1 Structure of the digital health research ecosystem

The bibliometric results demonstrate a highly collaborative and internationally connected research ecosystem. The prominence of authors, institutions, and countries from both African and Global North contexts highlights the central role of cross-national partnerships in shaping digital health scholarship. This pattern aligns with findings from comparable bibliometric analyses of digital health in LMIC regions, where North–South institutional partnerships have been consistently identified as a structural feature of the research ecosystem (37, 38).

Countries such as South Africa, Uganda, Kenya, and Ethiopia have emerged as important regional contributors, reflecting the gradual strengthening of local research capacity and institutional leadership. At the same time, institutions from the United States and the United Kingdom remain influential through long-standing collaborative research programmes and global health initiatives.

This hybrid research ecosystem indicates a gradual shift in which African institutions increasingly act as active producers of knowledge rather than solely as implementation sites, reflecting broader trends in global health research capacity development. Several scholars have argued that this transition is essential for ensuring that digital health innovations remain contextually grounded and aligned with African health system priorities rather than driven primarily by external research agendas (3, 33, 39).

### 4.2 Thematic structure of digital health research

The science-mapping analysis revealed several thematic clusters that together represent the intellectual structure of implementation-oriented digital health research in the selected African countries. Author keywords were used to create the thematic map rather than the database's indexing terms, to reduce thematic artefacts and increase thematic specificity.

The mobile health research theme is among the most mature, as shown by the thematic map. The cluster “mobile health–antenatal care–mobile phone” is especially situated in the quadrant of the motor-theme and has high centrality and density. This indicates a level of maturity in the field of maternal and antenatal mHealth interventions, conceptual integration, and its centrality in the broader digital health literature. The visibility of this cluster reflects the continued focus on maternal health care delivery, communications technologies, and mobile–enhanced care coordination in African health systems. This visibility aligns with systematic evidence that mobile health (mHealth) tools for antenatal care have shown substantial impacts on uptake of maternal services in sub-Saharan Africa contexts (40, 41).

The “mHealth–HIV–Kenya” cluster is found in the basic-theme quadrant, where there are relatively high centralities and comparatively low densities. The positioning suggests strong linkages with the wider research field and a relative lack of conceptual consolidation and intra-cluster development to HIV-care-based mHealth interventions compared to the maternal-health-based mobile health cluster. The results indicate that although mHealth interventions for HIV are extensively researched and have been implemented in practice, there is a need for more consistent theory building and implementation of frameworks to support the accumulation of learning in this field. Prior reviews of mHealth-based HIV interventions in Africa have also shown variation in outcome measures and theoretical underpinnings across studies, impeding the advancement of cumulative knowledge (4, 42, 43).

Themes related to “maternal health–digital health–Tanzania” are also present in the basic theme quadrant, indicating they are quite general but not well developed. This trend suggests further growth in digital maternal health research in healthcare implementation contexts. The cluster “telemedicine–public health–implementation”, situated in the niche-theme quadrant, had high internal cohesion but low connection with the thematic cluster. This indicates that telemedicine research is rather a specific field, with methodological and implementation-oriented research still concentrated there, rather than a part of the broader digital health research sphere. Themes of infrastructure deficiencies, regulatory hurdles, and staff readiness are also common findings in systematic reviews of telemedicine expansion in sub-Saharan Africa (8, 39).

Similarly, the “tuberculosis–adherence–Africa” cluster falls into the emerging or declining quadrant, indicating relatively low centrality and density within the thematic structure. While the focus of the cluster is tuberculosis, the literature also includes digital adherence interventions for TB, but this is a peripheral area, suggesting either a need to continue exploring this field or that it has yet to fit into the larger digital health research agenda. Research, therefore, needs to examine more interdependent cross-disease digital infrastructures that can connect tuberculosis management with HIV care, maternal health, and other primary healthcare delivery systems. This peripheral positioning is notable given established systematic review evidence on the effectiveness of digital tuberculosis adherence tools, suggesting this evidence base remains insufficiently integrated within the broader digital health research network (44).

### 4.3 Implications for digital health research and implementation

First, mobile health interventions were common across many disease areas, highlighting the need for greater conceptual integration across research disciplines. The position of the mHealth–HIV–Kenya cluster in the basic theme quadrant of the thematic map in Figure 8 suggests that, while mHealth is being broadly implemented across the field, the interventions are conceptually underdeveloped and lack integration with the theoretical frameworks. This trend highlights the need for digital health research to integrate behavioural science, health systems research, and implementation science findings to better understand how digital solutions can lead to enduring improvements in health system performance rather than isolated, intervention-specific evidence. This need is widely recognised in the global digital health literature, with scholars calling for theory-driven implementation frameworks to strengthen the cumulative evidence base for mHealth interventions (42, 43, 45).

Second, the trend-topic analysis (Figure 6) shows a clear shift in the time of the research from feasibility-oriented research in the first years of the study period (2015–2016) to a transition into implementation-oriented research and disease-specific digital applications from 2017 onwards. The shift reflects increasing awareness that digital health innovations need to be assessed in a real-world healthcare setting, not a controlled pilot setting. In line with this trend, the keyword frequency analysis (Figure 7) reveals that the term mHealth doubled from six mentions in 2015 to 127 in 2025, indicating a solid entry as a key implementation tool. To explore the potential effectiveness of digital interventions, research should focus on the system level, beyond individual-outcome measures, and look at how digital interventions interact with the organisational processes, workforce structure and governance frameworks in national health systems.

Third, the growing diversity of research themes throughout the study period is consistent with the thematic map's organization (Figure 8), which outlines telemedicine–public health–implementation as a niche theme with high internal thematic consistency and relatively few links to the other research clusters. The parallel positioning of tuberculosis–adherence–Africa in the emerging-or-declining quadrant further reinforces that disease-specific digital health initiatives remain structurally siloed despite years of research activity. The keyword co-occurrence network (Figure 9) presents two distinct thematic clusters, one implementation-oriented, the other methodological, with only a few connections between them. These patterns indicate that, in Africa, digital health research is growing in volume but remains largely not being integrated in a cross-domain thematic manner that would support the patient-centric and life-course approaches to healthcare delivery, as called for in global digital health policy frameworks (2, 3, 24, 39, 40, 44, 46).

### 4.4 Addressing the research questions

The findings of this study provide clear responses to the three research questions guiding the analysis. First, regarding RQ1, the performance analysis reveals a highly collaborative and globally interconnected research ecosystem. The United States, South Africa, the United Kingdom, and several East and West African countries, among those included in the geographic search block, emerge as leading contributors to implementation-focused digital health research. It should be noted that this ranking is bounded by the ten African countries named in the search strategy, and contributions from other nations, such as Egypt, Morocco, Senegal, and the DRC, are not directly reflected. Institutions such as the University of Washington, Makerere University, and the University of the Witwatersrand occupy central positions within the research network, highlighting the importance of sustained institutional capacity and international collaboration in advancing digital health scholarship.

Second, addressing RQ2, the thematic analysis identifies several dominant research clusters that structure the field. Mobile-health supported maternal and antenatal care, mHealth interventions in the delivery of HIV services, telemedicine and public-health implementation research, and tuberculosis-focused digital adherence interventions. The thematic distribution of these clusters suggests that digital health research in the selected African countries is increasingly focused on the delivery of care, implementation, integrated service platforms, and scalable mobile health systems.

Third, in response to RQ3, the thematic mapping and keyword network analyses reveal several emerging and underexplored areas that may shape future research. These include the development of interoperable digital health ecosystems, the expansion of digital platforms across multiple disease domains, and the integration of digital health tools within broader health system governance and service delivery structures.

### 4.5 Comparison with existing literature

The findings of this study align with and extend existing scholarship on digital health research in low- and middle-income countries, particularly in African health systems. Previous bibliometric and systematic analyses have highlighted the increasingly globalised nature of digital health research, characterised by strong international collaborations and the growing involvement of African research institutions (20–22, 26–31, 35–38, 40, 44, 46, 47). Consistent with these observations, the present analysis demonstrates that digital health research in Africa is embedded within an extensive global knowledge network in which institutions from the United States, the United Kingdom, and African countries collaborate closely. However, it is important to acknowledge that this network reflects a subset of implementation-focused research anchored in the countries included in the geographic search block, and the full scope of African digital health collaboration may be broader than this dataset captures. The prominent role of universities such as the University of Washington, Makerere University, and the University of the Witwatersrand reflects broader trends in global health research, where long-standing institutional partnerships and research consortia play a central role in advancing digital health innovation (20, 33, 37, 38).

The dominance of mHealth interventions identified in this study also reflects well-documented trends in the digital health literature. Several studies have shown that mobile health technologies have been widely adopted across African health systems to support treatment adherence, patient monitoring, and health communication, particularly in HIV care and maternal health services (8, 32, 41, 48). Reviews of mHealth implementation in sub-Saharan Africa similarly report that SMS-based reminders, mobile applications, and remote monitoring tools have significantly improved patient engagement and treatment adherence in infectious disease programmes (19, 39). However, as noted in previous research, the rapid expansion of these interventions has often occurred without strong theoretical integration across behavioural, implementation, and health systems frameworks, resulting in fragmented conceptual development within the field (42, 43).

The thematic clusters identified in this study further confirm that digital health research in Africa remains strongly oriented towards infectious disease management and maternal health, which have historically been priority areas for global health investments and digital innovation programmes (3, 32). This focus mirrors broader global health trends, in which digital tools are frequently implemented within vertical disease programmes, particularly those addressing HIV, tuberculosis, and maternal and child health outcomes. While these interventions have generated substantial evidence regarding effectiveness and feasibility, scholars have increasingly emphasised the need to move beyond disease-specific digital health initiatives towards integrated digital health systems capable of supporting multiple health conditions and care pathways (45, 49).

Finally, the identification of emerging research gaps in areas such as cross-disease digital infrastructures, integrated care platforms, and equity-focused digital health interventions corresponds with growing calls in global digital health policy and research agendas. International organisations and recent scholarly reviews have emphasised the need for digital health ecosystems that support coordinated care across disease domains while addressing persistent inequalities in access to digital technologies and healthcare services (2, 9, 10). In this context, the present study contributes new empirical evidence by demonstrating how digital health research in Africa is evolving towards more integrated, system-level approaches, while still facing important challenges related to theoretical consolidation and cross-disciplinary integration.

Taken together, the comparison with existing literature indicates that digital health research in Africa has reached a stage of rapid expansion and methodological consolidation, yet the field continues to face significant challenges in achieving conceptual integration and system-wide implementation. Addressing these challenges will be essential for ensuring that digital health innovations contribute effectively to sustainable and equitable health system strengthening across African contexts.

### 4.6 Future research directions

The following five research priorities are directly derived from the thematic patterns, keyword dynamics, and structural gaps identified in this study's bibliometric analysis:

#### 4.6.1 Theory-driven integration of digital health research

The mHealth–HIV–Kenya cluster is located in the basic-theme quadrant of Figure 8, with a high centrality but low study density, suggesting a general trend of widespread use of mHealth interventions without coherent theoretical underpinnings across studies. This fragmentation is also reflected in the keyword co-occurrence network (Figure 9), where there are weak connections between the implementation and methodological keyword clusters, as well as between implementation and health systems theory. This underscores the need for conceptual frameworks that can foster a more cumulative evidence base for mHealth and telemedicine research, which combine behavioural science and implementation science with health systems theory. The absence of such frameworks has been identified as a key barrier to the scalability of mHealth interventions in African health systems (42, 43).

#### 4.6.2 Shift from intervention efficacy to system-level effectiveness

Trend-topic analysis (Figure 6) revealed that feasibility and validation studies at early stages of development were predominant in the literature from 2015 to 2016, followed by implementation studies from 2017 onward. Even with this shift, however, the thematic map (Figure 8) shows that currently there is no cluster in the motor-theme position related to health system outcomes or sustainability evaluation, indicating that the field has not fully internalised system-level thinking, as it is embedded in its dominant research approaches. There is a need to go beyond a short-term pilot evaluation and use adaptive trials, comparative effectiveness designs, and approaches that incorporate real-world evidence and link individual patient outcomes to health system performance measures. Further, it is important to measure the long-term effects of digital health interventions beyond the initial implementation period; the bibliometric record is limited on long-term evidence, and if early gains in the use of mHealth-supported care are found to lead to health system long-term improvement, it will be vital to evidence-informed policy and investment decisions.

#### 4.6.3 Standardisation of outcomes and evaluation frameworks

The descriptive analysis (Table 1) shows that 1,170 unique author keywords were identified across 440 publications, suggesting that the terminology and conceptual framing across the studies are diverse. The lack of standardised outcome measures and reporting conventions across the field is illustrated by this terminological diversity, as evidenced by the patchy distribution of keyword co-occurrence, as shown in Figure 9. The lack of harmonised evaluation frameworks makes cross-study comparison and systematic evidence synthesis challenging, hindering the field's capacity to make evidence-based recommendations for action at the policy and implementation levels. The development of common outcome measures, such as for mHealth adherence and telemedicine delivery models, would significantly advance the body of evidence. This gap has been acknowledged in the global digital health literature, with the mHealth Evidence Reporting and Assessment checklist representing an early effort to standardise reporting, though consistent uptake remains limited (10).

#### 4.6.4 Development of interoperable digital care ecosystems

The niche theme quadrant, as displayed in Figure 8, is occupied by the telemedicine–public health–implementation thematic area, which has high internal coherence but limited linkage with other thematic areas, such as maternal health and HIV-related mHealth. The two relatively independent clusters in the keyword co-occurrence network (Figure 9) support the parallel evolution of telemedicine and mHealth research streams, with no clear links to integrated care models. Research should focus on the design and testing of interoperable digital platforms that integrate mobile health applications, telemedicine services, electronic health records and clinical decision-support systems into cohesive health delivery systems that can enable coordinated, cross-disease care.

#### 4.6.5 Integrated and equity-oriented digital health systems

The tuberculosis–adherence–Africa cluster is in the emerging-or-declining quadrant of Figure 8, with low centrality and low density, despite years of digital adherence work in this area. Such a peripheral positioning suggests that work on tuberculosis and digital health is still, to an extent, structurally separate from the thematic core of the field, which focuses on HIV and maternal health. Likewise, Table 6 reveals that there is no precision medicine or rare disease thematic cluster in the retrieved corpus, highlighting a substantial lack of equity in digital health research coverage across disease domains. Research should build on the current work on cross-disease digital infrastructures and explore sustainability, scalability, and the equity implications of digital health interventions across various populations, health conditions, and African healthcare settings, with a particular focus on conditions currently under-represented in the implementation-focused literature. Without deliberate attention to equity in digital health design and access, evidence suggests that digital innovations risk reinforcing rather than reducing existing health inequalities across diverse African populations (2, 39, 49).

### 4.7 Limitations

This study has several limitations that should be considered when interpreting the findings. First, although the study was based in Africa, the analysis was limited to English-language peer-reviewed journal articles in major databases, given the study's focus and direction. Digital health research in other African languages published in those languages, including implementation studies and regional articles, was not captured. Future bibliometric analyses should include multilingual search methods and a broader range of data sources to support more equitable and comprehensive representation across linguistically and institutionally diverse health systems in Africa.

Second, bibliometric methods primarily rely on metadata such as titles, abstracts, keywords, and citations. These are good indicators of publication trends, the type of collaboration and thematic patterns, but cannot measure the quality, effectiveness, or impact of digital health interventions in the real world. Third, the country-level findings are a boundary condition of the geographic scope of the search strategy. Selected African countries, South Africa, Kenya, Uganda, Nigeria, Ethiopia, Ghana, Tanzania, and Rwanda, were explicitly named in the Boolean search, as were more general terms like “Africa” and “Sub-Saharan Africa.” However, several countries with active digital health and health systems research programmes, including Egypt, Morocco, Senegal, Zimbabwe, Malawi, Cameroon, and the Democratic Republic of Congo, were not explicitly represented in the geographic search block. It is possible that publications from these countries that do not also refer to one of the selected countries in their title, abstract, or keywords were not included in the dataset. This may lead to a misrepresentation of the overall diversity of the digital health implementation research spectrum at the African country-level, as well as in the formation of collaboration networks and thematic patterns. Fourth, citation-based indicators are more likely to underestimate the impact of newer and emerging fields, such as artificial intelligence-assisted decision support, digital mental health, and interoperability frameworks. Fifth, the study was limited to digital health literature oriented to implementation, with inclusion criteria specifying both implementation- and care-setting- and outcome-related concepts in the search strategy. The search strategy used the general term ‘precision medicine’ but did not use more specific terminology related to rare diseases and genomics, such as ‘orphan disease’, ’sickle cell disease’, ‘pharmacogenomics’, ‘genomic medicine’ and ‘next-generation sequencing’. The absence of key terms in the retrieved corpus, therefore, is not indicative of a lack of digital health activity related to precision medicine or rare diseases in African contexts, but rather to the extent of the search design. A more complete understanding of the emerging field of rare-disease and genomic medicine research will, however, require the use of dedicated search strategies that incorporate explicit terms related to the field. Finally, the study uses a descriptive and exploratory analytical framework focused on mapping the evolution and thematic structuring of the literature and does not seek to establish causal relationships between digital health interventions and health system outcomes.

## 5 Conclusion

This study provides one of the most comprehensive bibliometric syntheses of digital health research across selected African countries between 2015 and 2025, offering a systematic overview of the field's intellectual structure, collaboration networks, and thematic evolution. By combining performance analysis with science-mapping techniques, the study reveals a rapidly expanding and increasingly collaborative research landscape centred primarily on maternal health, infectious disease management, and mobile health interventions. The findings demonstrate that digital health research in Africa has evolved significantly over the past decade. Early research largely focused on technology feasibility and pilot implementations, whereas more recent studies emphasise implementation science, health system integration, and outcome-oriented evaluations. This transition reflects the broader maturation of the field as digital technologies move from experimental tools towards core components of health system strengthening.

The analysis also highlights the central role of mHealth interventions, particularly in HIV and tuberculosis management and maternal health services, while identifying telemedicine and integrated digital platforms as emerging areas of research. At the same time, the thematic structure reveals important gaps, including limited conceptual integration across studies, insufficient cross-disease digital infrastructures, and the underrepresentation of certain health domains such as rare diseases and mental health. From a policy perspective, these findings underscore the importance of developing interoperable, scalable, and equity-oriented digital health ecosystems that support integrated care delivery across diverse health conditions. Strengthening digital health research capacity within African institutions, fostering sustainable international collaboration, and promoting standardised evaluation frameworks will be essential for translating digital innovation into long-term improvements in health system performance. By integrating performance analysis with science-mapping techniques, this study offers one of the first comprehensive bibliometric mappings of digital health research across multiple disease domains within African contexts.

Overall, implementation-focused digital health research across the studied African countries has reached rapid expansion and methodological maturity. However, significant gaps remain in theoretical integration, cross-disease digital infrastructures, and sustainable implementation strategies. The field has reached an inflexion point: the evidence base exists, but theoretical consolidation, systems-level integration, and longitudinal evaluation needed for equitable health system transformation are underdeveloped. Addressing this requires continued investment and stronger cross-sector collaboration guided by locally grounded implementation science frameworks. This study provides an evidence-informed roadmap for advancing equitable, interoperable, and resilient digital health systems across African contexts.

## Data Availability

Publicly available datasets were analyzed in this study. This data is available in the Scopus, PubMed, and Web of Science databases, as specified in the supplementary appendix. The processed dataset and scripts are available on reasonable request.
