# Supplementary material for: Digital health implementation research across selected African countries: a bibliometric analysis of maternal health and infectious diseases with observations on precision medicine representation (2015–2025)
Source: Front Digit Health. 2026 Jun 23;8:1831353. doi: 10.3389/fdgth.2026.1831353 (PMC13337819; doi:10.3389/fdgth.2026.1831353)
Supplement: Supplementary file 1 [file Supplementaryfile1.docx]

**Digital Health Implementation Research Across Selected African Countries: A Bibliometric Analysis of Maternal Health and Infectious Diseases with Observations on Precision Medicine Representation (2015–2025)**

**Appendix**

**Table A1.** Full database-specific search strings used for literature retrieval from Scopus, PubMed, and Web of Science Core Collection, with the number of records retrieved before screening and deduplication**.**

| Scopus | Articles |
| --- | --- |
| (TITLE-ABS-KEY(  "digital health" OR telemedicine OR tele-health OR mhealth  OR "mobile health" OR "clinical decision support"  )  AND  TITLE-ABS-KEY(  Africa OR "Sub-Saharan Africa" OR Kenya OR Nigeria OR Ghana OR Rwanda  OR "South Africa" OR Ethiopia OR Tanzania OR Uganda  )  AND  TITLE-ABS-KEY(  "maternal health" OR "maternal care" OR antenatal OR postnatal  OR "child health" OR neonatal  OR HIV OR malaria OR tuberculosis  OR "precision medicine"  )  AND  TITLE-ABS-KEY(  implement* OR deploy* OR evaluat* OR adoption  )  AND  TITLE-ABS-KEY(  hospital* OR clinic* OR "primary health care"  OR "community health worker*" OR CHW  )  AND  TITLE-ABS-KEY(  outcome* OR impact OR effectiveness OR uptake  )) | 340 |
| PubMed |  |
| (  ("digital health"[Title/Abstract] OR telemedicine[Title/Abstract] OR tele-health[Title/Abstract]  OR mhealth[Title/Abstract] OR "mobile health"[Title/Abstract]  OR "clinical decision support"[Title/Abstract])  AND  (Africa[Title/Abstract] OR "Sub-Saharan Africa"[Title/Abstract]  OR Kenya[Title/Abstract] OR Nigeria[Title/Abstract] OR Ghana[Title/Abstract]  OR Rwanda[Title/Abstract] OR "South Africa"[Title/Abstract]  OR Ethiopia[Title/Abstract] OR Tanzania[Title/Abstract] OR Uganda[Title/Abstract])  AND  ("maternal health"[Title/Abstract] OR "maternal care"[Title/Abstract]  OR antenatal[Title/Abstract] OR postnatal[Title/Abstract]  OR "child health"[Title/Abstract] OR neonatal[Title/Abstract]  OR HIV[Title/Abstract] OR malaria[Title/Abstract] OR tuberculosis[Title/Abstract]  OR "precision medicine"[Title/Abstract])  AND  (implement*[Title/Abstract] OR deploy*[Title/Abstract]  OR evaluat*[Title/Abstract] OR adoption[Title/Abstract])  AND  (hospital*[Title/Abstract] OR clinic*[Title/Abstract]  OR "primary health care"[Title/Abstract]  OR "community health worker*"[Title/Abstract] OR CHW[Title/Abstract])  AND  (outcome*[Title/Abstract] OR impact[Title/Abstract]  OR effectiveness[Title/Abstract] OR uptake[Title/Abstract])  ) | 231 |
| Web of Science |  |
| TS=(  ("digital health" OR telemedicine OR tele-health OR mhealth OR "mobile health"  OR "clinical decision support")  AND  (Africa OR "Sub-Saharan Africa" OR Kenya OR Nigeria OR Ghana OR Rwanda  OR "South Africa" OR Ethiopia OR Tanzania OR Uganda)  AND  ("maternal health" OR "maternal care" OR antenatal OR postnatal  OR "child health" OR neonatal  OR HIV OR malaria OR tuberculosis  OR "precision medicine")  AND  (implement* OR deploy* OR evaluat* OR adoption)  AND  (hospital* OR clinic* OR "primary health care"  OR "community health worker*" OR CHW)  AND  (outcome* OR impact OR effectiveness OR uptake)  ) | 331 |
